# Microbial Composition Change and Heavy Metal Accumulation in Response to Organic Fertilization Reduction in Greenhouse Soil

**DOI:** 10.3390/microorganisms13010203

**Published:** 2025-01-18

**Authors:** Qin Qin, Jun Wang, Lijuan Sun, Shiyan Yang, Yafei Sun, Yong Xue

**Affiliations:** 1 Institute of Eco-Environment and Plant Protection, Shanghai Academy of Agricultural Sciences, Shanghai 201403, China; qinqin19870987@126.com (Q.Q.); junwang2018@163.com (J.W.); sunliuliu@126.com (L.S.); 20220703@saas.sh.cn (S.Y.); 2 Key Laboratory of Low-Carbon Green Agriculture, Ministry of Agriculture and Rural Affairs, Shanghai 201403, China; 3 Shanghai Key Laboratory of Protected Horticultural Technology, Shanghai Academy of Agricultural Sciences, Shanghai 201403, China

**Keywords:** organic manure application, soil physicochemical characteristics, heavy metal, microbial community composition, greenhouse soil

## Abstract

Increased application of organic fertilizer is an effective measure to improve greenhouse soil quality. However, prolonged and intensive application of organic manure has caused nutrient and certain heavy metal accumulation in greenhouse soil. Therefore, the optimal quantity of organic manure required to sustain soil fertility while mitigating the accumulation of heavy metals and other nutrients resulting from continuous application remains unclear. This study evaluated the impacts of sustained and reduced organic manure application on soil physicochemical properties, heavy metal contents, and microbial community through a 9-year greenhouse field experiment. Treatments included a control without any fertilizer (CK), conventional manure (M), and three reduced manure treatments (−25%M, −37.5%MNPK, and −50%MNPK). Compared to CK, either M treatment or manure reduction treatments either maintained or significantly elevated soil pH and soil organic matter, total nitrogen, total phosphorus, and available phosphorus. Notably, −37.5%MNPK exhibited further increases in the available nitrogen and potassium. The M treatment significantly increased in the total concentrations of cadmium, copper, lead, zinc, and the availability of chromium and zinc. However, reduced manure treatments showed no change or a significantly reduced in heavy metal availability. The −25%M and −37.5%MNPK treatments significantly improved bacterial diversity. Reducing organic manure altered microbial taxa abundance. The soil pH emerged as the primary driving factor for variation in the bacterial community structure, whereas available nitrogen, potassium, and lead were the key factors influencing fungal community structural changes. These results indicate that reducing excessive organic manure input is an effective strategy to control heavy metal accumulation, enhance soil fertility, and optimize microbial community structure.

## 1. Instruction

Greenhouse crop production has rapidly developed in China and has become an important economic source in some locations. In pursuit of greater economic benefits, the percentage of fertilization increases annually to improve soil production and maximize crop yields [1]. From 1980 to 2019, the application of chemical fertilizers increased by 3.26 times in China. The negative effect on the system from continuous intensive application of chemical fertilization is substantial since it can greatly accelerate soil quality deterioration (i.e., soil acidification and salinization) and groundwater contamination.

Organic fertilizers like organic manures were increasingly recommended as an effective and sustainable practice to reduce these negative effects while bolstering soil fertility and quality [2,3,4,5]. However, farmers often resort to heavy applications of organic manure to maintain soil productivity, particularly when reducing the reliance on chemical fertilizer. The intensive and prolonged application of organic residues from poultry or swine farms poses a concern regarding heavy metal accumulation, as these animals are frequently fed supplements laden with metals such as cadmium (Cd), chromium (Cr), copper (Cu), lead (Pb), and zinc (Zn) [3,6,7]. Chen et al. [7] and Tian et al. [8] found that excessive manure application exacerbates the continuous accumulation of specific heavy metals (i.e., Cd, Pb, and Zn) in greenhouse soils, potentially impacting the whole greenhouse agro-ecosystem adversely. Meantime, over-fertilization of the greenhouse system can also result in the accumulation or surplus of nutrients (i.e., nitrogen, phosphorus, and potassium) in the soil [3,9,10], leading to runoff as a significant non-point source of groundwater pollution [10,11,12]. Thus, managing organics input to the soil for nutrient and heavy metal control is necessary to maintain greenhouse crop production and protect the agroecological environment.

Soil-dwelling microorganisms are pivotal in sustaining soil fertility and serve as valuable ecological barometers for assessing soil vitality [13,14]. These microbial assemblages exhibit heightened sensitivity to shifts in fertilization strategies, which can influence soil microbial ecosystems by altering the soil environment such as nutrient availability, pH, and metal contents [15,16]. For instance, inputs of organic manures either alone or in combination with chemical fertilizers could enhance available phosphorus and pH in the soil, fostering a diversification of microbial species, notably bacteria and fungi [13]. Moreover, Tong et al. [16] found a notable increase in fungal community abundance within small macro-aggregates upon organic manure application, which could further facilitate soil aggregation and carbon sequestration. Nevertheless, metal accumulation caused by excessive organic manure application could decrease soil microbial populations and alter the fungal-to-bacterial ratio [17]. This in turn disrupted the intricate metal–soil–plant interaction and decoupled the biogeochemical cycles of essential elements [18,19]. Recently, Duan et al. [20] documented that a reduction in organic manure application decreased metal contents (such as Cd) in the surface soil of the greenhouse. However, the specific responses of greenhouse soil microorganisms to decreased organic manure inputs remain unclear. Therefore, elucidating how microbial communities and the soil environment fluctuate in response to various organic manure fertilization techniques is crucial for devising strategies that optimize manure application, thereby enhancing greenhouse soil production while minimizing environmental pollution.

In this study, we assessed the impacts of various amounts of manure, alone or in conjunction with chemical fertilizers, on soil fertility, heavy metal accumulation, and the composition of soil microbial assemblages in a long-term field experiment conducted within a greenhouse. Our objectives were (1) to determine whether reducing manure application could mitigate heavy metal accumulation while potentially causing a concurrent decrement in soil nutrient availability and microbial diversity; (2) to examine the interconnections among soil microbiota, soil fertilization, and heavy metal contents; and (3) to propose fertilization strategies for the sustainable greenhouse crop cultivation while minimizing environmental contamination.

## 2. Material and Methods

### 2.1. Experimental Site and Design

The field experiment was undertaken within the greenhouse at the Zhuanghang Comprehensive Experiment Station (30°53′34″ N, 121°23′33″ E) of the Shanghai Academy of Agricultural Sciences, Shanghai, China. The experimental site is located in a subtropical marine monsoon climate, characterized by an annual precipitation of 1247 mm (with an evaporation rate of 1273 mm) and an average temperature of 17.3 °C. The soil profile at the experimental site, specifically the 0–20 cm layer, was identified as Fluvent with a sandy loam texture. Prior to initiating the long-term field experiment, soil analysis revealed a pH of 7.21, along with concentrations of 18.90 g kg^−1^ soil organic matter (SOM), 19.0 g kg^−1^ total nitrogen (TN), 0.77 g kg^−1^ total phosphorus (TP), and 15.63 g kg^−1^ total potassium (TK). Furthermore, the soil contained 63.24 mg kg^−1^ of available nitrogen (AN), 86.88 mg kg^−1^ of available phosphorus (AP), and 80.00 mg kg^−1^ of available potassium (AK). The field experiment, which began in September 2014, focused on continuous watermelon cultivation (one time per year) and incorporated five fertilizer treatments, i.e., no fertilizer (CK), a conventional application of organic manure resulting from local farming practices (M), and three reduced organic manure treatments. Specifically, the reduced treatments involved a 25% reduced rate on the basis of conventional rate (−25%M); a 37.5% reduced rate on the basis of conventional rate plus chemical N, P, and K fertilizer (−37.5%MNPK); and a 50% reduced rate plus chemical N, P, and K fertilizer (−50%MNPK). The chemical fertilizers were administered in the forms of urea (containing 46% N), potassium sulfate (52% K_2_O), and superphosphate (12% P_2_O_5_) fertilizer. Organic manure was completely composted swine manure (40% moisture) with pH of 6.37 and TN, TP, and TK of 16.1, 19.7, and 19.1 g kg^−1^, respectively. The contents of heavy metals in the organic manure were as follows: Cd 0.15 mg kg^−1^, Cr 10.6 mg kg^−1^, Cu 33.49 mg kg^−1^, Pb 4.9 mg kg^−1^, and Zn 134.96 mg kg^−1^. Prior to watermelon sowing, organic manure and chemical K fertilizer served as base fertilizers. Chemical N and P fertilizers were split into base and topdressing applications. The applied rates of all fertilizers are detailed in Table 1. The experiment layout adhered to a randomized block design with three replicates. Each treatment plot measured 4 m by 5.5 m, and plots were separated by a 0.4 m deep concrete barrier to minimize cross-contamination.

### 2.2. Soil Sampling and Analyses

Surface soil samples (0–20 cm) were sampled from five random locations in each plot after watermelon harvest and meticulously combined to form a composite sample. These freshly obtained soil samples were promptly transported to the laboratory, where they were subsequently divided into two distinct subsets. One subset was preserved at −80 °C for analyzing microbial characteristics, whereas the other subset was air-dried to enable the analysis of physicochemical characteristics and metal concentrations. Soil pH was measured with a pH meter in a 1:2.5 (*w*/*v*) soil/distilled water suspension. SOM was determined via dichromate oxidation and titration with ferrous sulfate [21]. TN and AN were measured via the Kjeldahl digestion method and alkali hydrolyzation, respectively [22]. TP and AP were, respectively, extracted with NaOH and NaHCO_3_ solution, followed by measurements via Mo-Sb colorimetric method [23]. AK was measured via the ammonium acetate method [24]. TK and the total contents of Cd, Cr, Cu, Pb, and Zn were measured via inductively coupled plasma mass spectrophotometry (ICP-MS, Agilent 7900, Santa Clara, CA, USA) after a digestion process involving nitric acid (HNO_3_), hydrochloric acid (HCl), hydrofluoric acid (HF), and perchloric acid (HClO_4_). The available heavy metals in the soils were extracted using diethylene triamine pentaacetic acid (DTPA) at a pH of 7.3 and a solid/liquid ratio of 1:2 (*w*/*v*) for a duration of 2 h, and the extractant was then analyzed for Cd, Cr, Cu, Pb, and Zn via graphite furnace atomic absorption spectrometry (280Z, Agilent, Santa Clara, CA, USA) [25].

### 2.3. DNA Extraction, Gene Amplification and Sequencing

DNA isolation was carried out from 0.25 g soil sample utilizing the FastDNA Spin Kit specially designed for soil specimens (MP Biomedicals, Santa Ana, CA, USA). The extraction procedure adhered strictly to the manufacturer’s guidelines. The quantity and purity of the DNA extracts were determined using a NanoDrop 2000 spectrophotometer (Thermo Scientific, Wilmington, NC, USA). The isolated DNA samples were then stored at −80 °C for future analysis.

The amplification of the V3–V4 region of the bacterial 16S rRNA gene via PCR was conducted in 20 µL reaction mixtures comprising 4 µL of 5 × FastPfu buffer (TransGen, Beijing, China), 2 µL (2.5 mM) of dNTPs, 0.8 µL (5 µM) each of primers 338F (5′-ACTCCTACGGGAGGCAGCAG-3′) and 806R (5′-GGACTACHVGGGTWTCTAAT-3′), 0.4 µL of FastPfu DNA Polymerase, and about 10 ng of template DNA. Similarly, the fungi ITS1 region was amplified in 20 µL reaction mixtures comprising 2 µL of 10 × Buffer, 2 µL of dNTPs at 2.5 mM, 0.8 µL (5 µM) each of primers ITS1F (5′-CTTGGTCATTTAGAGGAAGTAA-3′) and ITS2R (5′-GCTGCGTTCTTCATCGATGC-3′), 0.2 µL of rTap Polymerase, and approximately 10 ng of template DNA. The PCR protocol was executed in triplicate under the following conditions: an initial denaturation step at 95 °C for 3 min, followed by 35 cycles of denaturation at 95 °C for 30 s, annealing at 55 °C for 30 s, extension at 72 °C for 45 s, a final extension at 72 °C for 10 min, and termination at 10 °C. The triplicate PCR products for the same sample were combined and purified using the AxyPrep DNA Gel Extraction Kit sourced from Axygen Biosciences, Los Angeles, CA, USA. The purified PCR products were then pooled in equimolar ratios and subjected to paired-end sequencing on an Illumina MiSeq PE300 platform (Illumina, San Diego, CA, USA), adhering to standard protocols provided by Majorbio Bio-Pharm Technology Co., Ltd. (Shanghai, China).

The microbial sequences underwent analysis utilizing the Quantitative Insights into Microbial Ecology (QIIME) software package (version 1.9.1). To achieve high-quality clean reads, low-quality sequences were meticulously filtered under precise criteria, employing the Fastp quality-controlled process (version 0.19.6, https://github.com/OpenGene/fastp (accessed on 26 January 2019)) [26]. These filtered sequences were then merged using FLASH software (version 1.2.11) [27]. Subsequently, the quality-assured sequences were grouped into operational taxonomic units (OTUs) based on a 97% similarity threshold, facilitated by UPARSE (version 7.1). Post-clustering, representative sequences were chosen after rigorous removal of potential chimeras, according to Edgar et al. [28]. Taxonomy assignments of 16S rRNA OTUs were determined at a 70% threshold using the Ribosomal Database Project (RDP) Classifier (version 2.2) against the SILVA (version 138, https://www.arb-silva.de/ (accessed on 16 December 2019)) database [29], although UNIT database (version 7.2, https://unite.ut.ee/ (accessed on 18 November 2018)) was used for ITS OTUs [30].

### 2.4. Statistical Analysis

A statistical approach utilizing a one-way ANOVA was implemented, followed by Student–Newman–Keuls multiple comparisons tests at a significant level of *p* < 0.05, to assess the impact of fertilization on various soil attributes, including physicochemical properties, heavy metal concentrations, and microbial community compositions. These analyses were executed by the SPSS 21.0 software. All results were presented as average values with standard deviations. To investigate the Bray–Curtis dissimilarity of bacterial and fungal communities in soil samples subjected to diverse organic fertilization treatments, Non-Metric Multidimensional Scale (NMDS) analysis was conducted. The statistical significance of these results was validated using ANOISM with 999 permutations. Furthermore, Least Discriminant Analysis (LDA) effect size measurements were conducted to identify significant taxonomic variations between the treatments with an LDA score threshold of 2.0. The non-parametric factorial Kruskal–Wallis sum-rank test, employing an all-against-all comparison strategy, was used to pinpoint significant differences (*p* < 0.05). Taxonomic diagrams were crafted to depict the distinct taxa contributing to the observed differences between treatments. Additionally, Redundancy Analysis (RDA) (Canoco 5.0) and Pearson’s correlation analysis were applied to explore the relationships among soil properties, heavy metal concentrations, and microbial community compositions. Structural equation modeling (SEM), utilizing AMOS 26.0 software (IBM SPSS Amos) with the generalized least squares (GLS) estimation method, was employed to evaluate the direct and indirect effects of reduced manure application on these soil parameters. All graphical representations in the paper were created using OriginPro 2022 software.

## 3. Results

### 3.1. Physicochemical Properties

After prolonged application, notable increments in soil pH were evident under both M and −25% M treatments, with respective increases of 0.31 and 0.32 units compared to the CK treatment (Table 2). Interestingly, no significant difference in soil pH was discerned between the M and −25%M treatments; in other words, a 25% reduction in the conventional manure application rate could also prevent soil acidification. Under −37.5%MNPK and −50%MNPK, the soil pH showed no significant change relative to the CK, indicating that no further acidification occurred after 37.5% and 50% chemical fertilizer addition.

Unlike the trend of soil pH, SOM contents increased under all manure treatments to 17.94–21.61 mg kg^−1^, but the increasing extent reduced as the manure application rates decreased (Table 2). Soil TN and TP concentrations were always higher and significantly increased (except TN at −50%MNPK) for all manure treatments relative to the CK (Table 2) but unchanged in TK under any of the treatments. Notably, the −37.5%MNPK treatment exhibited the highest soil AN content, which surpassed that of the CK and M treatments by 1.30 and 1.25 times, respectively. Furthermore, soil AP and AK were significantly enhanced in all manure reduction treatments, with the −37.5%MNPK treatment boasting the highest soil AP and AK contents compared to the other two organic manure reduction treatments.

### 3.2. Total and Available Heavy Metals

The total concentrations of Cd, Cu, Pb, and Zn in the soil exhibited a notable increase under M by 1.12, 1.20, 1.10, and 1.16 times, respectively, in contrast to the CK treatment, with no significant change for total Cr contents (Table 3). The −25%M treatment significantly reduced total Cr and Cd concentrations in the soil by 8.12% and 22.30% in comparison to the CK treatment and 14.65% and 30.32% in comparison to the M treatment. Moreover, −37.5%MNPK and −50%MNPK increased total Pb concentrations in the soil (especially for −50% MNPK) by 8.75–11.25% compared to the CK treatment, whereas no statistically significant increments were detected for Cd, Cr, Cu, and Zn under these treatments.

Like the trend of the total concentrations of heavy metals, the available Cd (ACd), Cu (ACu), and Cr (ACr) in soil demonstrated a significant decline as the manure application rates reduced, relative to the CK. Conversely, no remarkable alterations were detected in available Pb (APb) and available Zn (AZn) (Table 4). Increasing chemical addition did not significantly increase their available contents (Table 4).

### 3.3. Microbial Community Composition and Diversity

From the soil samples analysis (a total of 15 samples), approximately 783, 260 and 758, 829 high-quality sequences were obtained, respectively, for bacteria and fungi, with respective average sequence lengths of 417.21 bp and 241.98 bp.

**Figure 1:**
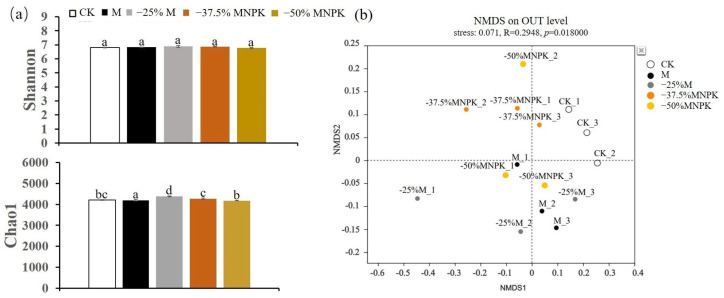
Soil bacterial richness and diversity under different organic manure treatments in the greenhouse system (**a**). Effect of organic manure reduction on bacterial community structure (β-diversity) analyzed by NMDS based on the Bray–Curtis distance matrix (**b**). Different lowercase letters represent a significant difference at *p* < 0.05.

**Figure 2:**
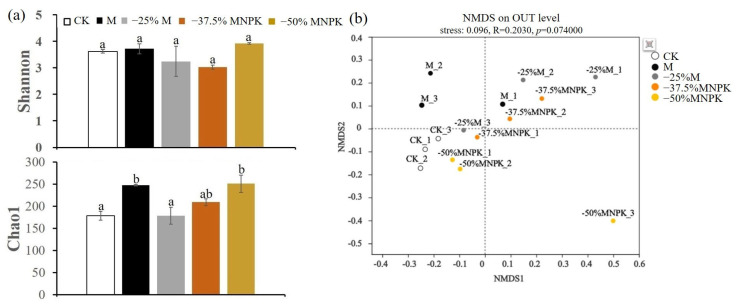
Soil fungal richness and diversity under different organic manure treatments in the greenhouse system (**a**). Effect of organic manure reduction on fungal community structure (β-diversity) analyzed by NMDS based on the Bray–Curtis distance matrix (**b**). Different lowercase letters represent a significant difference at *p* < 0.05.

The Shannon index was employed as the diversity index, and the richness index was estimated by the Chao1 index. The larger the Shannon and Chao1 indices, the higher the microbial diversity and community richness. The bacterial communities in the organic manure reduction regimes did not show significant differences in the Shannon index relative to the CK and M treatments in terms of diversity, whereas the Chao 1 index demonstrated different degrees of increase in terms of richness (Figure 1a). Among the different treatments of organic manure reduction, no significant differences were observed in the Shannon index, but the Chao1 index exhibited a declining trend with increasing chemical fertilizer application (Figure 1a). For fungi, organic manure reduction regimes did not significantly alter the Shannon and Chao1 indices relative to the CK and M treatments (Figure 2a). NMDS ordinations further revealed that organic manure reduction had a more pronounced impact on the bacterial community structure (Stress = 0.071, *p* < 0.018) (Figure 1b), but its impact on the fungal community structure was comparatively slight (Stress = 0.096, *p* = 0.074) (Figure 2b).

**Figure 3:**
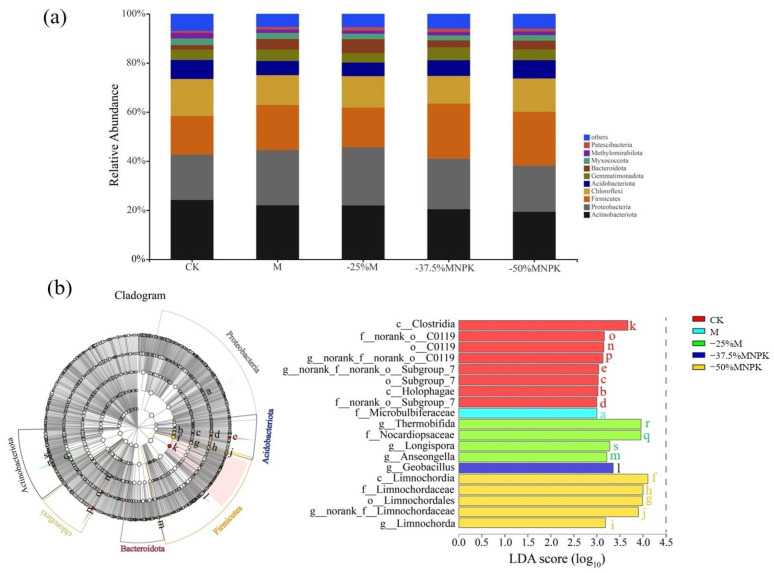
The relative abundance of soil bacterial community (at phylum level) under different organic manure treatments in the greenhouse system (**a**). LDA effect size (LEfSe) taxonomic cladogram of bacterial communities responding to different organic manure treatments (**b**). Significantly different taxon nodes are colored, and those that are not significantly different are non-colored. For each taxon detected, the corresponding node in the taxonomic cladogram is colored according to the highest-ranked group of the taxon. Different letters of the bar plot correspond to bacterial taxa for class, order, family, and genus in the pie chart.

In all samples, the bacterial community comprised in total 6581 OTUs, of which most were affiliated with Actinobacteriota (19.36–24.17%), followed by Proteobacteria (18.46–23.70%), Firmicutes (15.81–22.48%), and Chloroflexi (11.29–15.02%) (Figure 3a). These four phyla comprised over 73% of the entire bacterial community. Compared with the CK treatment, a significant reduction in the relative abundance of Actinobacteriota and Chloroflexi was observed, accompanied by an increase in the relative abundances of Proteobacteria and Firmicutes in soil samples. For organic manure-treated soil, no significant variations in bacterial relative abundance were observed as the applied amount of organic manure was reduced. However, LEFse revealed statistically significant differences in genus-level phylotypes across various organic manure reduction regimes (Figure 3b). For instance, *Thermobifida* and *Longispora* belonging to Actinobacteriota and *Anseongella* belonging to Bacteroidota in the −25%M-treated soil; *Geobacillus* belonging to Firmicutes in the −37.5%MNPK-treated soil; and *Limnochorda* belonging to the Firmicutes in the −50%MNPK-treated soil were more abundant (LAD > 3) but were less abundant in the M-treated soil (LAD > 3).

**Figure 4:**
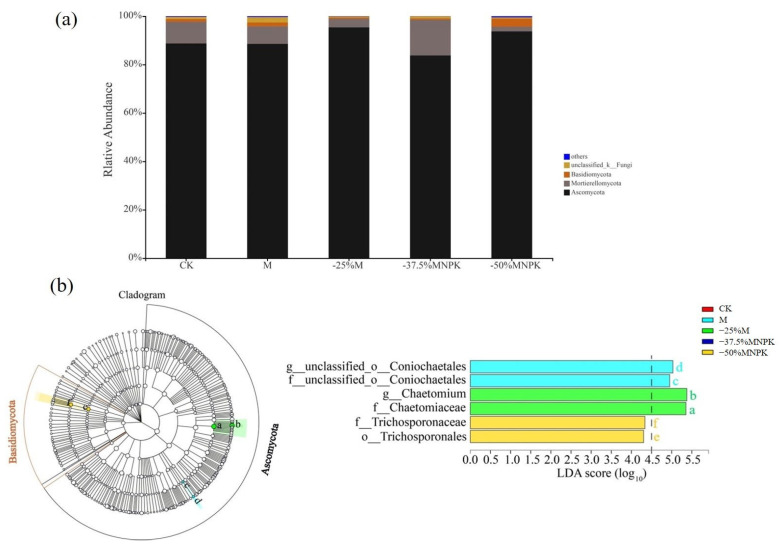
The relative abundance of soil fungal community (at phylum level) under different organic manure treatments in the greenhouse system (**a**). LDA effect size (LEfSe) taxonomic cladogram of fungal communities responding to different organic manure treatments (**b**). Significantly different taxon nodes are colored, and those that are not significantly different are non-colored. For each taxon detected, the corresponding node in the taxonomic cladogram is colored according to the highest-ranked group of the taxon. Different letters of the bar plot correspond to fungal taxa for order, family, and genus in the pie chart.

The fungal community clustered overall into 817 OTUs, which were assigned to three major phyla (Ascomycota, Mortierellomy, and Basidiomycota). Ascomycota emerged as the predominant phylum in the soils, accounting for 97.55–99.43% of the total fungal (Figure 4a). Across the soil samples, no significant change was observed in the Ascomycota relative abundance, but under −25%M and −50%MNPK treatments., the Ascomycota showed an increasing trend in comparison with the CK and M treatment. Utilizing LEFse analysis to identify distinct phylotypes at the genus level (LAD > 3), we found that *Chaetomium*, belonging to Ascomycota, was the enriched genus in the −25%M treatment, but no significant differences in genera were found for the −37.5%MNPK and −50%MNPK treatments, respectively, relative to the CK and M treatments (Figure 4b).

### 3.4. Relationship Between Soil Properties, Heavy Metals, and Microbial Composition

**Figure 5:**
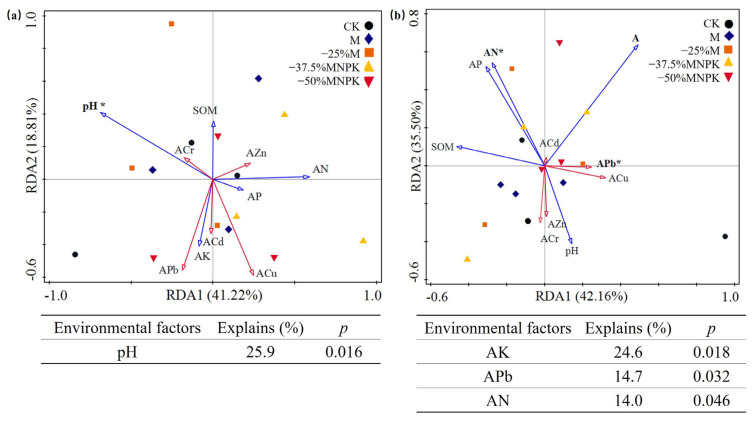
RDA plots showing the relationship between bacterial (**a**) and fungal (**b**) communities and ten environmental factors (SOM: Soil organic matter; AN: Available nitrogen; AP: Available phosphorus; AK: Available potassium; ACd: Available cadmium; ACr: Available chromium; ACu: Available cuprum; APb: Available plumbum; AZn: Available zinc). The statistically significant environmental factors and their explanations are shown below the plots, and the others are shown in Supplementary Table S1. The asterisk indicates the relationship between variances and microbial communities (* is significant at *p* < 0.05, without * is not significant at *p* > 0.05). (i.e., five soil properties including pH, AN, AP, AK, and SOM; five heavy metals including available Cd, Cr, Cu, Pb, and Zn).

The RDA/CCA analysis was conducted to quantify the relative contributions of soil properties (i.e., pH, AN, AP, AK, and SOM) and heavy metal (available Cd, Cr, Cu, Pb, and Zn) contents on the soil microbial community structure. The results indicated that the variance in the relationship between bacterial OTUs and environmental factors had a total of 60.03% contribution (Figure 5), and pH was the most significant variance that influenced bacterial communities. For fungal communities, the variable contribution was 77.66%, and AN was the most significant variance, followed by the soil AK and APb. However, the soil AP, SOM, ACd, ACr, ACu, and AZn had relatively less correspondence with bacterial and fungal communities, with the values of *p* > 0.05.

**Figure 6:**
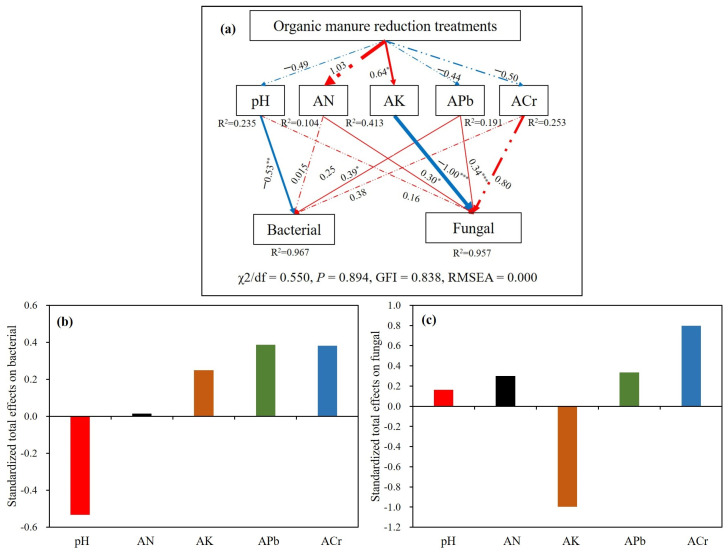
The structural equation model (SEM) showing the direct and indirect effects of the soil properties and soil heavy metals under different organic manure treatments on bacterial and fungal (**a**) and the standardized total effects (direct plus indirect effects) (**b**,**c**). The red arrows represent positive path coefficients, and the blue arrows represent negative path coefficients. R^2^ value shows how much of each variance can be explained. Continuous and dashed arrows indicate significant and insignificant relationships, respectively. Numbers beside the arrowed lines and the width of lines represent standardized path coefficients. The significant levels (Supplementary Table S2) at *p* < 0.05, *p* < 0.01, and *p* < 0.001 were denoted by *, **, and *** next to the number, respectively.

The structural equation model (SEM) provided good fits to the bacterial and fungal ratios, as evidenced by the χ^2^/df, *p,* and Goodness-of-Fit Index (GFI) metrics (Figure 6). The models explained 96.7 and 95.7% of the variances in bacterial and fungal ratios, respectively. The direct effects on bacterial were pH (path coefficients: −0.53, *p* < 0.01) and APb contents (path coefficients: 0.39; *p* < 0.05), whereas the direct effects on fungal included AN, AK, and APb contents (path coefficients: 0.30, −1.00 and 0.34; *p* < 0.05, *p* < 0.001 and *p* < 0.001, respectively). Additionally, organic manure reduction treatments had a positive and indirect association with bacterial via positively affecting AK contents and a negative and indirect effect on fungal via positively affecting AK contents. The standardized total effects on bacterial and fungal in organic manure reduction treatments were pH (−0.533) > APb (0.386) > ACr (0.382) > AK (0.249) > AN (0.015), and AK (−0.996) > ACr (0.796) > APb (0.335) > AN (0.299) > pH (0.163).

## 4. Discussion

### 4.1. Effect of Organic Manure Reduction on Soil Fertility

The sustained application of organic manure to the greenhouse led to an increase in soil pH (Table 2). This is primarily attributed to the inherently high pH and alkalinity of animal manures, which effectively counteract the protons generated during the process of nitrification [31,32]. Another important mechanism in increasing soil pH is the decarboxylation of organic anions in animal manures, which consumes protons and results in the release of CO_2_ [33]. However, when organic manure application with the conventional rates reduced by 25% (−25%M), soil pH still increased significantly in comparison with the CK treatment. This result indicated that the −25%M treatments provide sufficient alkalinity to neutralize the acidity produced by nitrification [3]. Thus, it is feasible to reduce organic manure rates and maintain greenhouse soil buffering capacity.

In this study, we also considered the hypothesis that adding chemical fertilizer to regulate the soil C/N ratio could more or less increase soil fertility. Diacono and Montemurro [34] analyzed overall studies and highlighted that the sustained application of organic manure alone had a beneficial effect on soil AK, extractable P, and organic carbon content, although it induced a retardation in the N availability process. Liu et al. [1] demonstrated that the combined application of organic manure and chemical fertilizer (i.e., 70% organic N and 30% inorganic N) was an effective strategy to enhance soil fertility. Similarly, our study found that −25%M treatment could also improve soil nutrient contents, but the effects were much weaker than with −37.5%MNPK and −50%MNPK. In particular, the −37.5%MNPK treatment did not significantly decrease soil pH and had higher soil available nutrient contents in comparison to the −50%MNPK treatment. The SOM content in −37.5%MNPK and −50%MNPK was also slightly increased; the reason was that balanced nutrition improvement improved the crop root exudate levels, which led to enhancing the SOM content [16]. However, the long-term application of organic manure alone (M and −25%M) primarily increased soil TN and TP contents in addition to SOM, which was not conducive to improving soil fertility. Therefore, to ensure that soil fertility does not limit greenhouse crop production, the appropriate addition of chemical fertilizer is necessary.

### 4.2. Effect of Organic Manure Reduction on Accumulation and Availability of Heavy Metal

The heavy metal elements of primary concern are Cd, Cr, Cu, Pb, and Zn in soil, which, when present in excessive amounts, can decrease greenhouse crop yields or degrade the quality of food produced [35]. The enclosed environment of the greenhouse, where crops are planted year-round, often leads to organic manure being the primary source of these heavy metals [36]. Hu et al. [37] showed that almost 98% of the total heavy metal inputs from manure sources could become plant-available after application. Our present study revealed that continuous and excessive application of organic manure significantly elevated the total concentrations of Cd, Cu, Pb, and Zn in comparison with the CK treatment (Table 3), which posed a risk for heavy metal contamination in greenhouse soil. This finding aligns with the previous study on a protected field with high manure application rates, where soil total Cd, Cu, and Zn concentrations surpassed their respective thresholds over a 15-year period [36]. Though the Cr content in organic manure was much greater than Cd and Pb contents, the total Cr contents in greenhouse soil did not increase significantly with the high application ratio of organic manure. This result should probably be attributed to the much higher background value of soil itself (at 64.6 mg kg^−1^) in comparison with Cd and Pb (at 0.134 and 21.3 mg kg^−1^, respectively). In this study, the total inputs of Cd, Cr, and Pb from swine manure were about 0.80, 3.83, and 0.024 kg ha^−1^ during the 9-year experiment with the M treatment, respectively. Therefore, total contents of Cd, Cr, and Pb would be increased by 1.24%, 17.99%, and 18.13% of background values, supposing those heavy metal elements remained in the soil (at 0–20 cm) without leaching or crop uptake. The estimation supports the remark increase in soil Cd and Pb but not Cr in the soil from swine manure application. Nevertheless, in the soil with a low background value of Cr, a trend of increased total Cr has been shown with increasing swine manure application rates [3].

To mitigate the risk of heavy metal pollution sourced from organic manure, the quality and quantity of organic manure applied (especially for the soil with low background values of heavy metal) must be determined. In this study, lower manure application indeed indicated a trend of decreased total concentrations of soil heavy metals, especially Cd and Cr. However, the −50%MNPK treatment significantly increased in soil TPb, indicating long-term chemical fertilizer input at this ratio may pose a risk of Pb pollution in greenhouse soil. Previous studies have shown that phosphate fertilizer input would lead to the accumulation of Cd and Pb in the soil. Thus, further investigation is needed to explore the interplay and the mechanism between P and total heavy metals sourced from phosphate fertilizers in the greenhouse.

The toxicity of soil heavy metals mainly depends on their available contents, rather than their total amounts. Our results showed that both organic manure reduction alone or combined with chemical fertilizer did not influence the available contents of Pb but significantly reduced the available contents of Cd, Cr, Cu, and Zn compared to conventional manure application rates (Table 4). This result indicated that organic manure addition played a more important role in increasing soil heavy metal availability in comparison with chemical fertilizer. Previous studies found that heavy metals present in organic manure had heightened bioavailability [38]. Therefore, reduced inputs of organic manure meant less SOM and less available heavy metal in the greenhouse soil. These results were the opposite of other studies on heavy metal contaminated soil, which showed substantial reductions in heavy metal availability following a high application rate of swine manure [39]. Organic matter has been documented to mitigate soil heavy metal bioavailability via adsorption or the formation of stable complexes with humus [40,41]. Nevertheless, organic matter can also function as chelators, enhancing heavy metal bioavailability and potentially introducing organic ligands [42]. Moreover, organic acid resulting from the decomposition of organic matter could mobilize strongly soil-sorbed heavy metals, thereby augmenting their release into the soil [38].

### 4.3. The Effect of Organic Manure Reduction on Microbial Community Structures

As soil heavy metal accumulation is caused by organic manure application, reducing the organic manure input and increasing the efficiency of organic matter utilization should be considered as part of the solution to soil heavy metal accumulation [32]. Soil microorganisms play a pivotal role in the process of SOC degradation and nutrient cycle. Fertilization practices significantly influence soil microbial composition and activity across various agricultural systems in the extent period [43].

To see how reducing organic manure input mitigates the impacts of fertilization on microbial communities and activity, Figure 1 and Figure 2 present a comparative analysis of the conventional and reduced organic amendment scenarios, both with and without chemical fertilization, in relation to the conventional organic manure (M) and unfertilized treatment (CK). The results indicated that a slight reduction in organic manure, coupled with a minor addition of chemical fertilizer, significantly altered the bacteria community composition in greenhouse soil (Figure 1). Specifically, treatments involving a 25% reduction in organic manure (−25%M) and those combining a 37.5% or 50% organic manure reduction with NPK fertilizer (−37.5%MNPK and −50%MNPK) exhibited higher bacteria species richness compared to the M and CK treatments. However, no profound effects on the diversity index were observed among these five treatments. It suggested that a low ratio of organic manure reduction (−25%M) or the combined application with low amounts of chemical fertilizer (−37.5%MNPK and −50%MNPK) did not reduce bacterial diversity but increased bacterial abundance and viability [44,45]. These results align with that of Han et al. [46], who found that organic manure incorporation with balanced NPK application positively influenced soil microbial activity by regulating nutrient supply. Similarly, Lazcano et al. [47] and Zainuddin et al. [48] found that biofertilizers or organic manure amended with a small amount of chemical fertilizer supplementation have significant positive effects on soil microbial activity and maintain nutrient levels comparable to organic fertilizer alone. Naher et al. [49] further noted that deficiencies in individual nutrient elements, especially N, might directly depress microorganism growth and reproduction. In this study, the −25%M and −37.5%MNPK applications maintained adequate nutrient supply for these heterotrophic bacteria, thus ensuring the growth and reproduction of bacterial communities.

In contrast to the bacterial community, fungal abundance was less affected by organic manure reduction treatments, particularly when chemical fertilizer was added. The main reason was attributed to the application of mineral N fertilizer, which typically promotes fungal growth [50]. This is consistent with Wang et al. [51], who reported significantly increased fungal richness with chemical fertilizer addition. However, fungal diversity and abundance were generally lower than those of bacterial communities under different treatments. Existing research suggests that fungi thrive better in acidic soils [52]. Given that the soil in our study was weakly alkaline (7.1–7.6), this would have significantly inhibited fungal growth.

An in-depth analysis of microbial community composition revealed that the integration of reduced organic manure with supplementary chemical fertilizer, notably at a rate of −37.5%MNPK, significantly improved the bacterial community structure, in agreement with prior studies [46,50]. The combined application of organic manure and chemical fertilizer enhanced the SOM contents and enriched them with essential nutrients like C, N, P, and K, providing optimal growth conditions for microorganisms and ultimately augmenting bacterial community diversity [46]. Intriguingly, our results indicated minimal variation in fungal community structures across different organic manure application strategies, inconsistent with existing studies. Specifically, Tong et al. [16] observed a significant increase in fungal gene abundance following organic fertilizer application, attributing this to SOC contents as a pivotal factor in the discrepancies. Conversely, other studies have documented a notable decrease in fungal gene abundances upon manure addition, which was attributed to the difference in soil type. Nevertheless, relevant research has also demonstrated that organic manure can elevate the proportion and biomass of fungi within the soil microbial community, which is inconsistent with our observations. The different results may stem from variations in methods, soil types, and environmental conditions. In contrast to natural ecosystems, the greenhouse environment produces a specific ecosystem because of its enclosed or partially enclosed roof, which elevated the air temperature, soil temperature, and soil moisture level in comparison with outdoor environments [16].

The incorporation of organic manure enhanced the relative abundance of *Alphaproteobacteria*, *Betaproteobacteria,* and *Gammaproteobacteria* [53], aligning with our results. The relative abundance of the predominant bacteria in the community, *Ptoteobacteria* and a copiotrophic bacteria with rapid growth and r-selected strategy, is often higher under nutrient-rich conditions with high C content [54]. The heightened levels of SOC in the organic manure amendments (M, −25%M, −37.5%MNPK, −50%MNPK) presumably fostered more conducive nutritional conditions for microbial proliferation, resulting in a higher abundance of this phylum compared to the no-fertilizer treatment. However, there were few significant variations in the predominant bacterial phyla abundances across the various organic manure treatments. The results implied that reducing organic manure application, whether alone or in conjunction with a balanced chemical fertilizer input, largely preserved the original relative abundance of soil bacteria. In addition, significant reductions in the relative abundance of *Actinobacteriota* were observed in all organic manure treatments. Previous studies have indicated that some members of *Actinobacteriota* can be considered K-strategists, exhibiting slower growth rates when resources consist of more intricate, recalcitrant organic materials. LEFse analyses highlighted a significant decline in the relative abundance of *C0119* and *Subgroup_7* in soil upon organic manure application. Recognizing that bacteria are pivotal decomposers in soil ecosystems, the long-term application of organic manure nurtured specific microbial populations that could efficiently utilize these nutrients [50], potentially sparking competition among *C0119*, *Subgroup_7*, and other bacterial species.

In terms of fungi, *Ascomycota* emerged as the dominant phylum in the agricultural soil [11,16], which is roughly similar to our research. Ascomycota are mostly terrestrial saprophytes and play critical roles in decomposing recalcitrant organic matter like cellulose, lignin, and keratin [55]. Based on the results of LEFse analysis, the incorporation of organic manure augmented the relative abundances of Ascomycota in the soil. This suggests that organic manure application is beneficial to increase nutrient cycling rates. However, no significant differences were observed in the relative abundances of the dominant fungal phylum in the soil between different organic fertilizer regimes, showing that even a reduced application of organic manure sustained a conducive environment for the growth and propagation of Ascomycota. An interesting deviation was observed with the −50%MNPK treatment, which showed an elevation in the relative abundance of the Basidiomycota phylum. A deeper analysis at the family level unveiled that *Trichosporonaceae* exhibited a notably higher abundance in the −50%MNPK treatment compared to other treatments (CK, M, −25%M, and −37.5%MNPK). This suggested that excessive chemical fertilizer inputs fueled the proliferation of *Trichosporonaceae*, potentially counteracting the beneficial effects of organic manure. Of concern, certain *Trichosporonaceae* species possess pathogenic traits or can synergistically enhance pathogenicity. Manure combined with appropriate inputs of chemical fertilizer appeared to mitigate the presence of *Trichosporonaceae*, potentially lowering disease incidence [56]. Bonanomi et al. [57] further underscored substantial variability in the disease-suppressing capabilities of organic amendments across different pathogens.

RDA revealed that soil pH emerged as the primary determinant of bacterial communities, whereas fungal communities were predominantly influenced by AN, AK, and APb. It indicated that shifts in soil pH of greenhouse soil might affect the bacterial community. This result is consistent with existing studies, which emphasized the pivotal role of soil pH in determining the bacterial compositions across diverse biogeographical scales and land-use types [11,58,59]. Specifically, He et al., [60] observed the highest numbers of ammonia-oxidizing bacteria in soil subjected to a sustained application of organic manure combined with mineral NPK fertilizer, whereas the lowest abundance was observed in the mineral N fertilizer treatment because of soil acidification. However, the specific relationships between bacterial taxa and soil pH varied among studies. For example, some studies observed a decline in Alphaproteobacteria abundance with increasing pH in black soil, Changbai Mountain soils, and tropical forest soils [58,61,62], whereas Kim et al. [59] found an increase up to pH 6.0–6.5 followed by a decrease in greenhouse soil. Similarly, Shen et al. [61] reported a decrease in Acidobacteria abundance with higher pH, but other studies found the abundance of this phylum had a positive correlation with soil pH [59]. These discrepancies might be attributed to differences in land-use types, such as greenhouse soil vs. mountain, forest, or pasture land [59]. Hence, further exploration of the correlation between soil pH and bacterial communities in greenhouse soil is imperative.

For the fungi, the variables AN and AK, not SOM, seemed to be the primary factors driving the community change in the greenhouse soil [63]. This indicated that organic manure application did not directly affect the fungal community but rather indirectly through enhanced nutrient transformation, thereby increasing the availability of N and K. The SEM further verified that organic manure reduction indirectly affected the fungi by altering AN and AK contents (Figure 6). Additionally, APb also emerged as an important factor affecting the fungal community in greenhouse soil, exerting a positive influence. Notably, in treatments with reduced organic manure (−25%M, −37.5%MNPK, and −50%MNPK), APb concentrations decreased, whereas AN and AK concentrations significantly increased. This could imply that the stimulatory effect of elevated nutrient availability on microbial diversity might compensate for the loss of microbial diversity due to decreased APb. Moreover, the relatively low fungal diversity observed suggested that fungal communities in greenhouse soil were shaped by the combined effects of multiple factors rather than by a single factor.

## 5. Conclusions

The incorporation of organic manure is regarded as an effective management strategy for enhancing soil properties, fostering microbial vitality, and alleviating heavy metal pollution in greenhouse soils. A comprehensive, long-term field investigation provided valuable data on the optimal management of organic manure amendment within the greenhouse soil. The results showed that a continuous high application of conventional organic manure indeed mitigated greenhouse soil acidification and increased the total soil nutrient contents. However, solely relying on continuous high organic manure application failed to maximize nutrient availability. Additionally, a notable challenge emerged, which was the substantial accumulation of total Cd, Cu, Pb, and Zn and available Cr and Zn, which could be mitigated by adjusting and reducing excessive organic manure input. To further augment soil nutrient availability, especially AN, supplementary chemical fertilizer application became necessary when excessive organic manure inputs were curbed. However, superabundant chemical fertilizer significantly elevated total Pb contents, ultimately contributing to Pb pollution. Furthermore, the biotic parameters of microorganism diversity, richness indexes, and composition were also significantly influenced by different fertilization practices. Notably, bacterial communication composition was more sensitive to the fertilization regime changes compared to soil fungi. The RDA showed that soil pH was the primary driver of bacterial community structural variation, whereas AN, AK, and APb were key factors of fungal community structural shifts. It underscores the complexity of the fungal community structure’s responses to fertilization practices, surpassing that of bacteria. In conclusion, our study revealed that the application of organic manure at a properly reduced rate, while applying an appropriate amount of chemical fertilizer (such as −37.5%MNPK), can reduce the accumulation and availability of some heavy metals, enhance soil fertility, and optimize the microbial community structure in greenhouse soil. However, a more comprehensive and detailed examination is imperative to thoroughly understand the interaction between fertilization practices and fungal communities in greenhouse soil to better guide the scientific utilization of organic manure and ensure the safe cultivation of greenhouse crops.

## Figures and Tables

**Table 1:** The applied rates (kg ha^−1^) of all fertilizers in the greenhouse system.

Treatment	Organic Manure (N-P_2_O_5_-K_2_O) *	Urea (N)	Potassium Sulfate (K_2_O)	Superphosphate (P_2_O_5_)
CK	/	/	/	/
M	18,000 (288-355-355)	/	/	/
−25%M	13,500 (216-266-266)	/	/	/
−37.5%MNPK	11,250 (180-222-222)	256 (133)	175 (112)	859 (103)
−50%MNPK	9,000 (144-177-177)	340 (177)	241 (154)	1142 (137)

* The values in the brackets were the nutrient contents converted from the total amount of organic manure or chemical fertilizer; / indicates no fertilizer has been added.

**Table 2:** Soil physicochemical characteristics under different organic manure treatments in the greenhouse system.

Treatment	pH	SOM (g kg^−1^)	TN (g kg^−1^)	TP (g kg^−1^)	TK (g kg^−1^)	AN (mg kg^−1^)	AP (mg kg^−1^)	AK (mg kg^−1^)
CK	7.30 ± 0.01 a	16.79 ± 1.87 a	0.97 ± 0.08 a	0.46 ± 0.01 a	19.90 ± 0.99 a	107.73 ± 2.67 a	22.95 ± 0.61 a	218.59 ± 24.29 a
M	7.61 ± 0.13 b	21.61 ± 0.23 c	1.38 ± 0.006 c	0.56 ± 0.01 b	20.30 ± 0.42 a	111.51 ± 2.67 a	37.26 ± 0.31 b	259.16 ± 52.98 a
−25%M	7.62 ± 0.01 b	19.77 ± 0.40 bc	1.34 ± 0.001 c	0.55 ± 0.03 b	19.85 ± 0.78 a	111.51 ± 2.67 a	36.07 ± 1.07 b	416.26 ± 46.01 b
−37.5%MNPK	7.28 ± 0.10 a	18.22 ± 0.49 ab	1.18 ± 0.04 b	0.55 ± 0.04 b	20.45 ± 1.06 a	139.86 ± 5.35 b	42.79 ± 3.83 c	467.79 ± 42.80 b
−50%MNPK	7.18 ± 0.01 a	17.94 ± 0.24 ab	1.07 ± 0.03 a	0.52 ± 0.01 b	20.50 ± 0.42 a	107.73 ± 13.36 a	32.92 ± 1.84 b	431.48 ± 65.61 b

Different lowercase letters in the same column represent a significant difference at *p* < 0.05.

**Table 3:** The total contents of heavy metals in greenhouse soil with different organic manure treatments.

Treatment	Cd (mg kg^−1^)	Cr (mg kg^−1^)	Cu (mg kg^−1^)	Pb (mg kg^−1^)	Zn (mg kg^−1^)
CK	0.139 ± 0.003 b	41.61 ± 1.67 c	24.95 ± 0.49 a	14.85 ± 0.06 a	85.96 ± 2.22 a
M	0.155 ± 0.008 c	44.79 ± 1.65 c	27.56 ± 1.00 b	17.79 ± 1.23 c	99.32 ± 4.30 b
−25%M	0.108 ± 0.004 a	38.23 ± 0.24 b	24.18 ± 0.84 a	14.69 ± 0.07 a	90.24 ± 0.08 a
−37.5%MNPK	0.147 ± 0.005 bc	37.73 ± 1.74 ab	25.45 ± 0.42 a	16.15 ± 0.33 ab	88.06 ± 3.12 a
−50%MNPK	0.142 ± 0.004 bc	34.64 ± 0.13 a	22.99 ± 0.91 a	16.52 ± 0.44 bc	92.70 ± 2.48 ab

Different lowercase letters in the same column represent a significant difference at *p* < 0.05.

**Table 4:** The available contents of heavy metals in greenhouse soil with different organic manure treatments.

Treatment	Cd (mg kg^−1^)	Cr (mg kg^−1^)	Cu (mg kg^−1^)	Pb (mg kg^−1^)	Zn (mg kg^−1^)
CK	0.054 ± 0.0040 b	0.0056 ± 0.000095 b	3.39 ± 0.077 c	1.96 ± 0.014 a	2.86 ± 0.31 a
M	0.055 ± 0.00073 b	0.0062 ± 0.000027 c	3.07 ± 0.099 b	2.15 ± 0.16 a	4.71 ± 0.30 b
−25%M	0.046 ± 0.0023 a	0.0051 ± 0.00014 a	2.63 ± 0.0099 a	1.86 ± 0.17 a	2.55 ± 0.049 a
−37.5%MNPK	0.053 ± 0.00023 ab	0.0052 ± 0.000034 a	3.34 ± 0.071 c	1.96 ± 0.012 a	2.59 ± 0.89 a
−50%MNPK	0.050 ± 0.0027 ab	0.0057 ± 0.000054 b	2.83 ± 0.15 ab	1.78 ± 0.11 a	2.87 ± 0.31 a

Different lowercase letters in the same column represent a significant difference at *p* < 0.05.

## Data Availability

The original contributions presented in the study are included in the article; further inquiries can be directed to the corresponding author.

## Supplementary Materials

The following supporting information can be downloaded at: https://www.mdpi.com/article/10.3390/microorganisms13010203/s1, Table S1: The individual explanatory rates of the environmental factors in RDA analysis; Table S2: The statistically significance of direct and indirect effects by SEM.
