# Supplementary material for: Microbial Composition Change and Heavy Metal Accumulation in Response to Organic Fertilization Reduction in Greenhouse Soil
Source: Microorganisms. 2025 Jan 18;13(1):203. doi: 10.3390/microorganisms13010203 (PMC11767376; doi:10.3390/microorganisms13010203)
Supplement: Supplementary file 1 [file microorganisms-13-00203-s001.zip › microorganisms-3421046-SI.pdf]

Table S1 The individual explanatory rates of the environmental factors in RDA analysis

Environmental factors	Bacterial		Fungal	
	Explains (%)	*P*	Explains (%)	*P*
pH	25.9	0.016	3.3	0.534
SOM	6.9	0.332	1.6	0.670
AN	2.8	0.732	14.0	0.046
AP	6.6	0.286	6.7	0.280
AK	3.9	0.536	24.6	0.018
ACd	5.0	0.446	7.6	0.106
ACr	3.1	0.670	3.0	0.572
ACu	1.7	0.852	8.0	0.114
APb	5.4	0.416	14.7	0.032
AZn	6.4	0.330	1.2	0.784

Table S2 The statistically significance of direct and indirect effects by SEM

Effect factors	Name	*P*
Organic manure reduction treatment	pH	0.234
Organic manure reduction treatment	AN	0.646
Organic manure reduction treatment	AK	0.044
Organic manure reduction treatment	APb	0.201
Organic manure reduction treatment	ACr	0.231
pH	Bacterial	0.020
AN	Bacterial	0.652
AK	Bacterial	0.169
APb	Bacterial	0.011
ACr	Bacterial	0.650
pH	Fungal	0.172
AN	Fungal	0.023
AK	Fungal	<0.001
APb	Fungal	<0.001
ACr	Fungal	0.567
